# Six minute walk distance and reference values in healthy Italian children: A cross-sectional study

**DOI:** 10.1371/journal.pone.0205792

**Published:** 2018-10-15

**Authors:** Matteo Vandoni, Luca Correale, Mariangela Valentina Puci, Christel Galvani, Roberto Codella, Fabio Togni, Antonio La Torre, Francesco Casolo, Alberto Passi, Claudio Orizio, Cristina Montomoli

**Affiliations:** 1 Laboratory of Adapted Motor Activity (LAMA), Department of Public Health, Experimental and Forensic Medicine, University of Pavia, Pavia, Italy; 2 Unit of Biostatistics and Clinical Epidemiology, Department of Public Health, Experimental and Forensic Medicine, University of Pavia, Pavia, Italy; 3 Applied Exercise Physiology Laboratory, Department of Psychology, Università Cattolica del Sacro Cuore, Milan, Italy; 4 School of Exercise Sciences, Department of Biomedical Sciences for Health, Università degli Studi di Milano, Milan, Italy; 5 Department of Clinical and Experimental Sciences, University of Brescia, Brescia, Italy; 6 Department of Pedagogy, Exercise and Sport Science Degree Course, Università Cattolica del Sacro Cuore, Milan, Italy; 7 Department of Medicine and Surgery, University of Insubria, Varese, Italy; 8 Metabolism Research Center, IRCCS Policlinico San Donato, San Donato Milanese, Italy; Universidade Federal da Bahia, BRAZIL

## Abstract

The 6-minute walking test (6MWT) is a simple assessment tool to evaluate exercise capacity. The result of the test is the distance that a subject can walk at a constant and normal pace within 6 minutes (6MWD) and reflects the aerobic/fitness performance related to walking function. Use of 6MWT has been relevant to assess exercise tolerance either in healthy children or in patients with, heart, lung and metabolic diseases. Our aim was to find 6MWT reference values in healthy Italian children. The 6MWT was performed in 5614 children aged 6–11 years recruited from primary Italian schools. Age related reference percentiles of the covered distance were gender-modeled. A linear and quadratic regression model was used to predict 6MWT performance. Males walked longer distances than females, respectively 598.8±83.9 m vs 592.1±77.6 m (p = 0.0016). According to the regression analysis, 6MWD was positively related to age, gender and height, while it was negatively related to body weight *[(6MWD = -160*.*16 + 93*.*35× age (years) -4*.*05× age*^*2*^ *(years) +7*.*34× gender (m) +2*.*12× weight (kg) −2*.*50× height (cm)]*. Reference values were established for the 6MWT in healthy children. The age related 6MWD percentiles provided a useful tool in the assessment of capacity in 6–11 year children, in fact they may be helpful to evaluate the effect of a given treatment or rehabilitation program and represent a feasible measure as to prevention within the primary school context. It was found a substantial difference from other countries for 6mwd values. In our study, factors such as age, weight and height were relevant for the prediction of 6MWD, similarly to other studies. Therefore, these variables should be taken into account in context of exercise performance.

## Introduction

The 6-minute walking test (6MWT) is a test used to asses exercise capacity. The test measures the distance that subject walked (6MWD) in a constant and normal pace within 6 minutes and it reflects endurance and walking function covered at a submaximal level [1].

Use of 6MWT in children has been relevant to assess exercise tolerance [2] in pathological conditions such as cardiovascular diseases, asthma, cystic fibrosis, end-stage renal disease and pulmonary hypertension [3–9]. This simple test has been increasingly used over the past decade in healthy children because it is quick, easy to administer, especially in an evaluation setting without specific instrumentations, inexpensive, well understood, accepted and tolerated and more reflective of daily living activities than other exercise tests [10].

Even though it is common that clinicians and researchers use reference values from foreign samples, values of 6MWD between countries are divergent. Different studies demonstrate that there is a large variability (up to 159 meters) in children of different nationalities [11]. Moreover, demographic and anthropometrics characteristics such as age, gender, height and weight could affect the performance of the test [10–21].

For these reasons, more detailed studies are needed to establish reference values in different countries’ populations. At the moment in Italy, there are no reference values of 6MWD among healthy children. The aim of this study is to establish reference distances values for 6MWT in healthy Italian children between 6–11 years and to investigate the influence of age and anthropometrics on the walked distance.

## Materials and methods

### Participants

This cross-sectional study involved children aged 6–11 years recruited from forty primary schools of Lombardy, participating at the Regional project “Lombardia in gioco: A Scuola di Sport” between November 2016 and May 2017. Children with known chronic cardiac, respiratory, neurological or musculoskeletal disorders were excluded, such as a recorded walked distance shorter than 300 or longer than 850 m.

### Human subjects approval statement

The study was approved by the university ethical board (University of Pavia, degree-course in Exercise and Sport Science) and by the institutional boards of the participating schools. Parents or legal guardians gave written informed consent for all enrolled children. All the procedures used complied with the principles of the Declaration of Helsinki.

### Procedure

Anthropometrics of the children were measured before the test sessions using standardized techniques. Height was measured using a portable stadiometer with a precision of ± 1mm, with children in an upright position, with bare feet placed slightly apart, arms extended and head positioned parallel to the floor. Body weight was assessed digitally or using a beam scale with a precision of ± 100g, with children in light clothing, without shoes, and stood upright at the center of the platform of the weight scale. Body Mass Index (BMI) was subsequently calculated by the equation *body weight (kg)/height*^*2*^ *(m*^*2*^*)*. We calculated the age of the children from birth date and subsequently rounded down values.

The 6MWT was performed according to the American Thoracic Society (ATS) guidelines [1]. Participants, after a 10-minute rest period, were instructed to walk as fast as possible without running or jogging and were allowed to stop whenever they wanted. Researchers encouraged the participants with standardized phrases, as described by ATS. The test was conducted in a flat, straight corridor with a hard surface. Each participant walked continuously for six minutes at a self-selected pace along a 20-m measured tape line, with cones placed at each end of the course. Evaluators explained the test procedures before the start. To ensure that the children understood the instructions, one practice trial over one track length was completed. During the real test, every child was followed by a ‘safety chaser’ giving limited standardized encouragements. Five kinesiologists who had received the same training during 3 specific sessions dedicated to the standardization of the test procedures, performed 6MWT.

### Statistical analysis

To describe the sample, we calculated summary statistics that are expressed as means and standard deviations or percentages, as appropriate. Data were tested for normality by Shapiro-Wilk tests and graphically checked for linearity. We used Student T test for independent data or corresponding non-parametric test (Mann-Whitney test) to compare the quantitative variables. Percentile curves for 6–11 years was performed with the LMS Chart Maker Pro version 2.43 software program (http://homepage.mac.com/tjcole). Percentile curves were constructed using the 3^rd^, 10^th^, 25^th^, 50^th^, 75^th^, 90^th^ and 97^th^ percentiles of the 6MWD for the male and female subjects. We conducted correlation analyses by two groups of age and applied the z-test using a Fisher-z transformation to test the difference between two correlation coefficients. To estimate a prediction equation for the 6MWD a multiple linear regression model was fitted. The residuals were plotted against fitted values. Interaction effect were analyzed. Performance and goodness of fit of the model were assessed using the root-mean-square error (RMSE) and R-squared measures. Statistical analysis was conducted using STATA/SE for Windows, version 12.1 (StataCorp, college Station, TX, U.S.A.). A p-value <0.05 was considered significant.

## Results

**Fig 1:**
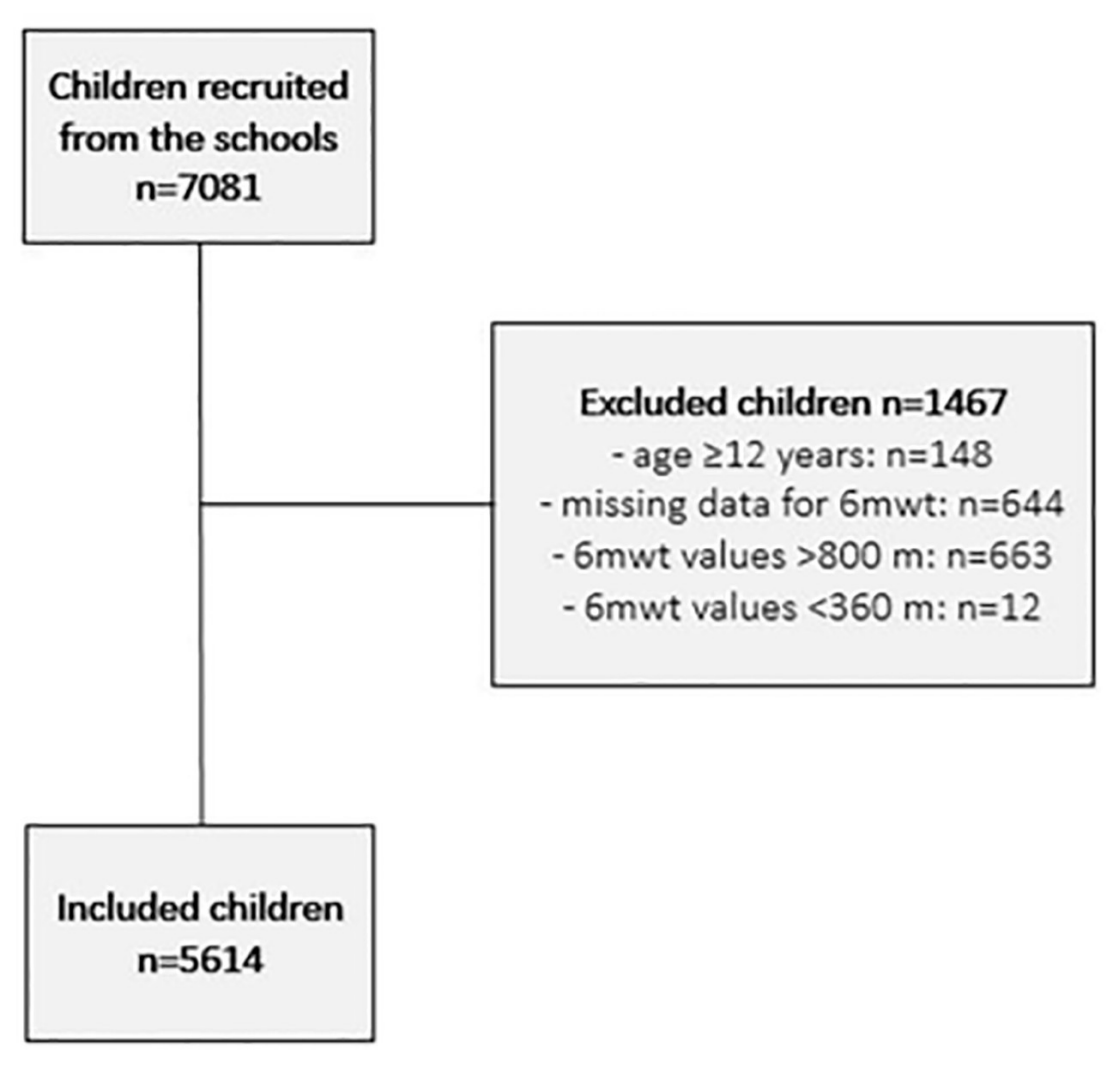
Flow chart showing study selection process.

A total of 5614 children were recruited for the study, 51% were males and mean age was 8.4 ± 1.5 years. Fig 1. shows study selection process.

### Demographic and anthropometric characteristics and 6MWT data

In males, mean 6MWD value was 598.8 ± 83.9 m and in females 592.1 ± 77.6 m (p = 0.0016). Table 1 shows anthropometric and demographic data and 6MWD values for all ages. Mean walked distance for all participants was 595.5 ± 80.9 m (range 360–800 m), values ranged from 513.3 ± 60.4 m for 6-year old children to 656.1 ± 71.6 m for 11-year old children. The 6MWD increased from 513.3 ± 60.4 m at 6 years to 656.1 ± 71.6 m, the walked distance was comparable until 8 years, then the velocity increased progressively. In 6–8 year old children, mean 6MWD value was 558.18 ± 72.32 m, while in 9–11 year old children were 634.84 ± 70.15 m (p<0.0001).

**Table 1:** Anthropometrics/demographic data and 6-minute walk distance in different age categories.

Age (years)	n	M/F	Height (cm)	Weight (kg)	BMI (Kg/m^2^)	6MWD (m)
6	687	350/337	116.7±5.0	22.1±3.9	16.1±2.0	513.3±60.4
7	1076	550/526	122.2±5.4	25.0±4.8	16.6±2.4	547.2±65.2
8	1115	577/538	127.9±5.7	28.2±6.0	17.1±2.8	596.5±65.8
9	1077	556/521	132.9±6.2	31.2±6.7	17.5±2.8	617.9±65.0
10	1139	551/588	139.6±6.5	36.2±8.2	18.4±3.2	641.1±70.6
11	520	262/258	143.4±6.6	39.0±9.1	18.8±3.4	656.1±71.6
Total	5614	2846/2768	130.3±10.3	30.0±8.5	17.4±2.9	595.5±80.9

Values are expressed by mean ± standard deviation.

S1 Table shows data for males. 6MWD data start from 515.6±65.6 m for 6 years old sample and reach 660.8±74.8 m for 11 years old sample. Mean value for the whole sample is 598.8±83.9 m. For females, 6MWD data start from 510.8 ± 54.4 m for 6 years old sample and reach 651.4 ± 68.1m for 11 years old sample. Mean value for the whole sample is 592.1 ± 77.6 m (S2 Table). Regarding height, S3 Table shows means of 6MWD for different height groups. Values ranged between 513.9 ± 63.0 m for children tall less than 114 m to 661.4±63.3 cm for children taller than 155 m.

Table 2 shows correlation coefficients between demographic/anthropometrics and 6MWD for the whole sample and for two age subgroups (6 to 8 years and 9 to 11 years). For all children, age, weight and height correlated significantly with the 6MWD (r = 0.58; r = 0.32; r = 0.52; p<0.05). Comparison between the two age categories revealed that, in younger children, correlations between 6MWD and height, weight, age and gender were significantly higher compared with older children (p<0.0001).

**Table 2:** Correlation coefficients between 6MWD and anthropometrics/demographic data for the total group and for two age subgroups.

	Total group	Age subgroups		z-value	p-value
				For age subgroups comparison	
	6–11 years	6–8 years	9–11 years		
n	5614	2878	2736		
Age	0.58*	0.52*	0.23*	12.41	p<0.0001
Weight (kg)	0.32*	0.19*	0.02	6.45	p<0.0001
Height (cm)	0.52*	0.40*	0.20*	8.27	p<0.0001

*p<0.05.

### Percentile curves according to age by male and female

**Fig 2:**
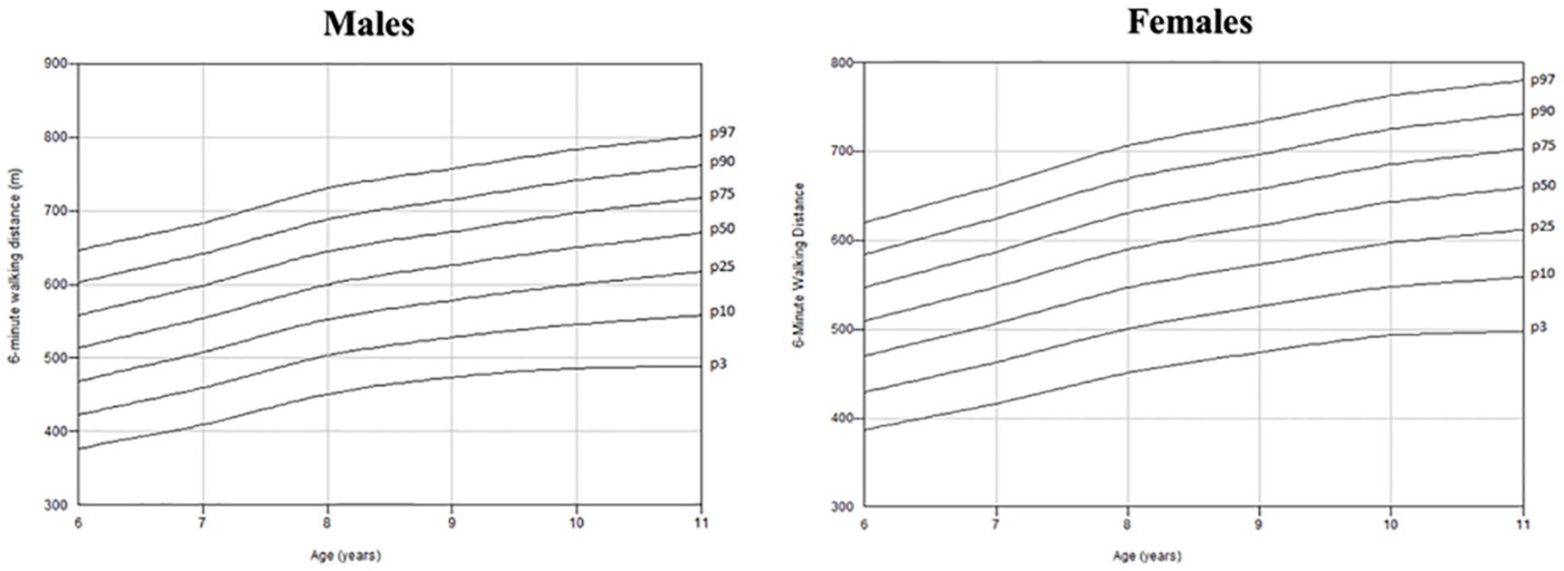
Normal reference centile charts for 6-minute walking distance in males and females.

Fig 2 shows the percentiles range from 3^rd^ to 97^th^, by gender for different values of age.

For males, in order of age, the 50^th^ percentile values resulted in 511 m, 554 m, 603 m, 624 m, 649 m and 671 m. For females, the 50^th^ percentile values resulted in 512 m, 543 m, 597 m, 620 m, 643 m and 655 m (S4 Table).

### Predictors of 6MWD

The regression model showed that age, gender, weight and height had a significant predictive impact on the 6MWD. More precisely, age, gender and height were positively related to 6MWD, while weight was negatively related to 6MWD. The model accounted for 37% of the total variance, the RMSE was 64.17 m (Table 3). No interaction effects were statistically significant.

**Table 3:** Predicting model for walk distance (6MWD, n = 5614).

	Regressors	Standard Error	p-value	95% CI	
R^2^ = 0.37					
RMSE = 64.17 m					
p<0.0001					
Age	93.35	7.61	p<0.0001	78.43	108.27
Age^2^	-4.05	0.44	P<0.0001	-4.93	-3.18
Gender (males vs females)	7.34	1.72	p<0.0001	3.98	10.71
Weight (kg)	-2.12	0.17	p<0.0001	-2.45	-1.78
Height (cm)	2.50	0.19	p<0.0001	2.12	2.88

The following formula was obtained to predict the distance walked in the 6 min test: *[(6MWD = -160*.*16 + 93*.*35× age (years) -4*.*05× age*^*2*^ *(years) +7*.*34× gender (m) +2*.*12× weight (kg) −2*.*50× height (cm)]*. S5 Table shows the comparison between present and other studies’ results including measured and predicted values of 6MWD. Measured mean values of present study were similar to Belgium [17]. Our data were comparable to South American ones until 7 year of age, then there was a slightly increase of 6MWD values for Italian children of 8–11 years [22]. Overall, North African children [14] showed 6MWD higher values than Italian children. In contrast, results from English children were lower than the Italians ‘ones [16].

## Discussion

The aim of the present study was to establish reference values of the 6MWD in Italian primary school children. Age-adjusted percentile curves were obtained for both genders and could be useful to find an altered performance in functional capacity to exercise. As far as we know, this is the largest national and international dataset on 6MWD values for 6–11 years children [11, 19].

6MWT is a useful tool in the assessment of exercise tolerance in children of primary school, in our study all children performed equally well in the 6MWT and reported no difficulties such as tasks understanding and execution of the procedures. Moreover, 20-m distance seems to be advantageous for the younger children (6–8 years) because it allows them to more closely focus on the task. In addition, 20-m is a distance often available in school or pediatrician settings [10].

The variation in walked distance in children of 6–8 years was significantly different than in older children. As stated by Lammers et al. [16, 17], our results suggest the presence of a more rapid increase in the distance walked in smaller children with an added slower increase up to 11 years of age [16]. In accordance with the previous findings [10, 14, 16, 22], we found a moderate correlation between the 6MWD and age and height. In the age subgroups comparison, we observed that in younger children (6–8 years) the correlations between 6MWD and age and anthropometric data were higher than in older children, consistently with previous results from Austria [10], Britain [16] and Belgium [17].

In addition, the walking distance could be influenced by physical activity levels ([4]). This may explain the general observation that sedentary children tend to exercise less and have poorer results in 6mwd. Our primary school population was found to be active in sport practice (close to 60%) in post school time ([23]). These data could justify the good performance of our sample respect to other countries. Similarly to other populations [10, 16, 17, 22], after the age of 8 years, anthropometric characteristics impacted less on 6MWD, despite changes due to growth [10, 14, 16]. A possible explanation could be owed by the improvement in gait and muscle activation patterns that allows an estimated 5km/h speed in walking [24, 25] subsequent to the 8-year old age.

According to other studies [18, 20, 21, 26], we observed that males walked longer distances than females, for this reason age-related percentile curves for the 6MWD were developed for both gender. As expected, our study confirmed that demographic and anthropometric characteristics could influence the 6MWT performance in healthy subjects. In our model, age, gender, height, and weight, could predict the 6MWD. More accurately, 6MWT distance mainly depended on age. In addition, weight and height significantly add information and they should be taken into account in such a particular growth phase. It is not surprising, therefore that a taller stature was associated with a longer stride, which probably resulted in a longer distance walked by taller subjects. The observed gender discrepancy of the 6MWD may be explained by the typical differences of functional parameters during exercise between genders at this age.

Finally, values obtained by the regression model were similar and coherent with the results from other countries [10, 14, 17, 26]. We are aware that our study has some limitations, in fact it was not possible to collect other physiological outcomes (e.g. heart rate, blood pressure and oxygen saturation) since it was performed in a school setting with numerous classes which were hardly managed in a scheduled time table. Moreover, we are conscious that our sample come entirely from Lombardy Region and it could be a bias despite Lombardy is the most densely populated Region of the country(and sedentary level of its children (16%) is comparable to the National one (18%) [23].

In a future perspective it would be interesting to study more data involving physiological parameters, in view to compare exercise capacity more accurately in healthy children and in children with cardiovascular and respiratory diseases.

## Conclusions

This study has established data on reference values for the 6MWT in Italian healthy children. The 6MWD age-related percentiles provide a useful tool in the assessment of exercise capacity in children aged 6–11 years. In our study, factors such as age, weight and height were relevant for the prediction of 6MWD, similarly to other studies. Therefore, these variables should be taken into account in context of exercise performance. The test was feasible in the school’s setting even with a large sample of pupils. Given this premise, our study could be a starting point to deeper investigate Italian children 6MWD of other Regions. However, further research is required to identify additional factors influencing the 6MWT.

## Supporting information

S1 Table Demographic data and 6-minute walk distance of male in different age groups. (PDF)

S2 Table Demographic data and 6-minute walk distance of female in different age groups. (PDF)

S3 Table Anthropometrics/demographic data and 6-minute walk distance in different height groups. (PDF)

S4 Table 6 minute walking distance norms for Italian children. (PDF)

S5 Table Measured and predicted 6MWD of the present study and comparison with reported and predicted 6MWD. (PDF)
