# Correction: Six minute walk distance and reference values in healthy Italian children: A cross-sectional study

**DOI:** 10.1371/journal.pone.0208179

**Published:** 2018-11-21

**Authors:** Matteo Vandoni, Luca Correale, Mariangela Valentina Puci, Christel Galvani, Roberto Codella, Fabio Togni, Antonio La Torre, Francesco Casolo, Alberto Passi, Claudio Orizio, Cristina Montomoli

The affiliation for the fifth author is incorrect. Roberto Codella is not affiliated with #4–8 but with #4 and #8: School of Exercise Sciences, Department of Biomedical Sciences for Health, Università degli Studi di Milano, Milan, Italy and Metabolism Research Center, IRCCS Policlinico San Donato, San Donato Milanese, Italy.

## References

[1] Vandoni M, Correale L, Puci MV, Galvani C, Codella R, Togni F, et al (2018) Six minute walk distance and reference values in healthy Italian children: A cross-sectional study. PLoS ONE 13(10): e0205792 10.1371/journal.pone.0205792 30321226 PMC6188863

