# Supplementary material for: Six minute walk distance and reference values in healthy Italian children: A cross-sectional study
Source: PLoS One. 2018 Oct 15;13(10):e0205792. doi: 10.1371/journal.pone.0205792 (PMC6188863; doi:10.1371/journal.pone.0205792)
Supplement: S1 Table — (PDF) [file pone.0205792.s001.pdf]

## Supporting information

**S1 Table.** Demographic data and 6-minute walk distance of male in different age groups.

| Age (years) | n | Height (cm) | Weight (kg) | BMI ($Kg/m^2$) | 6MWD (m) |
|---|---|---|---|---|---|
| 6 | 352 | 117.1±5.1 | 22.2±4.2 | 16.1±2.1 | 515.6±65.6 |
| 7 | 553 | 122.9±5.4 | 25.4±4.9 | 16.8±2.4 | 550.0±69.4 |
| 8 | 577 | 128.7±5.7 | 28.9±6.3 | 17.3±2.9 | 602.1±67.2 |
| 9 | 557 | 133.4±6.3 | 31.8±6.8 | 17.8±2.8 | 623.0±67.1 |
| 10 | 558 | 139.5±6.4 | 36.3±8.5 | 18.5±3.4 | 643.1±75.7 |
| 11 | 267 | 143.2±6.2 | 38.9±8.5 | 18.9±3.2 | 660.8±74.8 |
| Total | 2864 | 130.5±10.0 | 30.3±8.5 | 17.5±3.0 | 598.8±83.9 |

Values are expressed by mean ± standard deviation.
