# Supplementary material for: Six minute walk distance and reference values in healthy Italian children: A cross-sectional study
Source: PLoS One. 2018 Oct 15;13(10):e0205792. doi: 10.1371/journal.pone.0205792 (PMC6188863; doi:10.1371/journal.pone.0205792)
Supplement: S2 Table — (PDF) [file pone.0205792.s002.pdf]

Supporting information

S2 Table. Demographic data and 6-minute walk distance of female in different age groups.

Age (years)	n	Height (cm)	Weight (kg)	BMI (Kg/m^2)	6MWD (m)
6	340	116.3±4.8	22.0±3.7	16.2±2.1	510.8±54.4
7	528	121.5±5.5	24.6±4.8	16.6±2.4	544.2±60.4
8	539	127.0±5.6	27.5±5.7	17.0±2.8	590.4±63.7
9	521	132.5±6.1	30.5±6.6	17.3±2.9	612.4±62.3
10	592	139.8±6.8	36.1±7.9	18.4±3.1	639.3±65.2
11	261	143.7±7.1	39.1±9.8	18.8±3.6	651.4±68.1
Total	2781	130.0±10.6	29.8±8.6	17.3±3.0	592.1±77.6

Values are expressed by mean ± standard deviation.
