# Supplementary material for: Six minute walk distance and reference values in healthy Italian children: A cross-sectional study
Source: PLoS One. 2018 Oct 15;13(10):e0205792. doi: 10.1371/journal.pone.0205792 (PMC6188863; doi:10.1371/journal.pone.0205792)
Supplement: S3 Table — (PDF) [file pone.0205792.s003.pdf]

Supporting information

S3 table. Anthropometrics/demographic data and 6-minute walk distance in different height groups.

Classes of height (cm)	n	Age (years)	Height (cm)	Weight (kg)	BMI (Kg/m^2)	6MWD (m)
<114	313	6.3±6.4	111.6±2.5	19.7±2.6	15.8±1.9	513.9±63.0
114-124	1421	7.0±0.8	120.0±2.8	23.4±3.3	16.2±2.1	546.1±69.8
124-134	1908	8.3±1.0	129.3±2.8	28.8±4.7	17.2±2.6	599.6±69.6
134-144	1439	9.6±0.8	138.9±2.8	35.4±6.6	18.3±3.2	635.6±71.0
144-155	490	10.2±0.5	148.0±2.5	43.2±8.0	19.7±3.5	651.9±68.3
>155	43	10.5±0.3	158.2±3.4	52.8±9.8	21.1±3.7	661.4±63.3

Values are expressed by mean ± standard deviation.
