# Supplementary material for: Six minute walk distance and reference values in healthy Italian children: A cross-sectional study
Source: PLoS One. 2018 Oct 15;13(10):e0205792. doi: 10.1371/journal.pone.0205792 (PMC6188863; doi:10.1371/journal.pone.0205792)
Supplement: S4 Table — (PDF) [file pone.0205792.s004.pdf]

Supporting information

Table S4. 6-minute walking distance norms for Italian children.

Male Percentile	6 years (n = 350)	7 years (n = 550)	8 years (n = 577)	9 years (n = 556)	10 years (n = 551)	11 years (n = 262)
3th	397.2	410	467.3	494.7	501.6	500.5
5th	405.6	424	488	508.5	517.2	520
10th	436.1	455.1	515.4	533.4	538	540.6
20th	461.2	494	548	565	574	598.4
25th	470	505.8	559	582	587	615.8
30th	480	516.3	567	590.1	602.6	639.4
40th	497	534.4	583	608	626	653.2
50th	511	554	603	624	649	670.8
60th	526.6	570	619	643.2	670	686
70th	545	588	640	658.5	690	711
75th	558.3	600	650	665	700	718
80th	569.6	610	660	682	713.6	722.8
90th	601.8	635.8	688	710	739.8	747.1
95th	628	660	710	729.3	760	763.4
97th	659.5	676.5	728.6	744.6	768.9	777.1

Female Percentile	6 years (n = 337)	7 years (n = 526)	8 years (n = 538)	9 years (n = 521)	10 years (n = 588)	11 years (n = 258)
3th	416.3	426.2	463.3	485	516	520
5th	422	440	483.9	504.2	531	529
10th	442.8	464	501.8	532.2	550	559.4
20th	460.6	497.4	535.8	560	581	583
25th	471	505	547.8	573.5	598	599
30th	480	512.1	556.4	582	605.7	616.7
40th	498	528.8	578	600	624.6	639.6

50th	512	543	597	620	643	655
60th	524	560	611	630.2	660	676.4
70th	538.6	580	625	647.4	676.8	694
75th	545	587	635	656.5	686.8	705.3
80th	553.8	593.8	643	665.6	699.2	714.2
90th	578.2	622	670.1	692	720	732.1
95th	600	640	694	705	738.3	753.1
97th	613.6	648.2	700.8	716.7	750	760.5

Values are expressed in meters.
