# Supplementary material for: Six minute walk distance and reference values in healthy Italian children: A cross-sectional study
Source: PLoS One. 2018 Oct 15;13(10):e0205792. doi: 10.1371/journal.pone.0205792 (PMC6188863; doi:10.1371/journal.pone.0205792)
Supplement: S5 Table — (PDF) [file pone.0205792.s005.pdf]

Supporting information

S5 Table. Measured and predicted 6MWD of the present study and comparison with reported and predicted 6MWD

	Age					
	6	7	8	9	10	11
Measured 6MWD						
Present study.	513.3±60.4	547.2±65.2	596.4±65.8	617.9±65.0	641.1±70.5	656.1±71.6
Goemans et al.[17]	516.1±61.8	559.2±65.4	604.3±72.0	595.7±69.0	633.1±70.0	625.9±83.0
Saad H Bem et al.[14]		543.0±33.0 (6-7 y)	667.0±55.0 (8-9 y)		715.0±31.0 (8-9 y)	
Geiger et al.[10]		577.8±56.1 (6-8 y)			672.8±71.6 (10-11 y)	
Klepper et al. [15]			534.5±60.3 (7-8 y)	515.8±81.4	497.9±74.0	534.9±88.9
Lammers et al.[16]	463.0±40.0	488.0±35.0	483.0±40.0	496.0±53.0	506.0±45.0	512.0±41.0
Ulrich et. al. (25)	535.0±73.0	603.0±51.0	596.0±59.0	627.0±70.0	655.0±53.0	624.0±87.0
De Assis Pereira Cacau et al.(18)		474.4±83.3	514.1±77.1	525.0±81.0	549.5±87.2	557.3±98.7
Priesnitz et al.[22]	508.3±54.0	550.2±61.6	556.7±67.2	594.2±60.6	602.4±61.1	608.0±54.3
Predicted 6MWD						
Present study.	510.4±13.1	550.5±17.0	590.5±15.7	620.6±15.1	643.8±15.3	654.5±15.4
Geiger et al.[10]	556.9±12.1	586.9±7.7	611.9±9.1	635.4±8.5	655.6±11.2	670.2±9.6
Saad H Bem et al.(14)	595.9±21.1	624.3±17.2	652.7±18.3	677.0±18.2	703.4±25.4	718.8±23.0

In Lammers et al. and in our study, mean distances are reported for males and females together. In all other studies, all measured are reported for males.
